# The C-Terminus of *Toxoplasma* RON2 Provides the Crucial Link between AMA1 and the Host-Associated Invasion Complex

**DOI:** 10.1371/journal.ppat.1001282

**Published:** 2011-02-10

**Authors:** Jessica S. Tyler, John C. Boothroyd

**Affiliations:** Department of Microbiology and Immunology, Stanford University School of Medicine, Stanford, California, United States of America; University of Geneva, Switzerland

## Abstract

Host cell invasion by apicomplexan parasites requires formation of the moving junction (MJ), a ring-like apposition between the parasite and host plasma membranes that the parasite migrates through during entry. The *Toxoplasma* MJ is a secreted complex including TgAMA1, a transmembrane protein on the parasite surface, and a complex of rhoptry neck proteins (TgRON2/4/5/8) described as host cell-associated. How these proteins connect the parasite and host cell has not previously been described. Here we show that TgRON2 localizes to the MJ and that two short segments flanking a hydrophobic stretch near its C-terminus (D3 and D4) independently associate with the ectodomain of TgAMA1. Pre-incubation of parasites with D3 (fused to glutathione S-transferase) dramatically reduces invasion but does not prevent injection of rhoptry bulb proteins. Hence, the entire C-terminal region of TgRON2 forms the crucial bridge between TgAMA1 and the rest of the MJ complex but this association is not required for rhoptry protein injection.

## Author Summary

Invasion by the obligate intracellular parasites, *Toxoplasma* and *Plasmodium*, requires the formation of a ring of contact between parasite and host plasma membranes, the so-called moving junction (MJ), that the parasite migrates through during entry. The MJ is a complex of secreted parasite proteins including AMA1, on the parasite surface, and several rhoptry neck proteins (RONs), which are reported to associate with the host plasma membrane. The precise nature of the interaction that causes these two membranes to be so tightly apposed has not yet been elucidated. Here we report that the carboxy-terminal region of *Toxoplasma* (Tg)RON2 is exposed to the extracytosolic face of the MJ and that two short domains (D3 and D4) within this region independently and efficiently interact with the exposed ectodomain of TgAMA1. As recombinant D3, representing just 54 amino acids from TgRON2, efficiently blocks invasion, this interaction represents the crucial linkage for the MJ complex. Interestingly, D3 does not prevent injection of a rhoptry reporter protein demonstrating that invasion, and specifically a functional MJ, is not required for such injection. Our results suggest that the D3–D4 subregion of RON2, which is conserved across the Apicomplexa, will be a potent addition to current, AMA1-based control strategies for malaria.

## Introduction

Protozoan parasites are a significant cause of morbidity and mortality in humans worldwide. Among the most devastating and globally prevalent parasites are the members of the phylum Apicomplexa, which includes the etiological agents of malaria, cryptosporidiosis, and toxoplasmosis. Apicomplexans are related by an anterior complex of specialized secretory organelles that secrete molecules necessary for active host cell invasion and subsequent development of the parasitophorous vacuole (PV) around the penetrating parasite [1]. Given the obligate intracellular nature of these organisms, invasion of host cells is a critical event in the host-parasite interaction.

In contrast to many intracellular pathogens that use conventional host-uptake pathways to enter a target cell, apicomplexans actively invade in a rapid, multi-step process that is dependent on the parasite actinomyosin machinery [2], [3]. A distinctive feature of this process is the formation of a close apposition between the parasite and host plasma membranes that is reminiscent of a tight junction in mammalian cells [4], [5]. Beginning with its apical end, the parasite moves through this ring-like structure which is referred to as the ‘moving junction’ (MJ) and which functions to generate the PV membrane from the invaginated host plasma membrane [6]. As invasion proceeds, the MJ also appears to act as a molecular sieve that somehow excludes certain host membrane proteins from the forming PV membrane [7].

The identified heteromultimeric protein complex that forms at the MJ is derived from two distinct secretory organelles of the parasite: the micronemes and the rhoptry neck compartment [8], [9], [10], [11], [12], [13], [14]. The micronemal protein AMA1, which has a type I transmembrane topology in the parasite plasma membrane, is the most well characterized molecule of the MJ complex. The importance of this apicomplexan-specific protein in the invasion process has been directly demonstrated in several members of the phylum, including *T. gondii* [15], [16], *Babesia bovis* [17], and *Plasmodium spp.* [18], [19]. In *Toxoplasma*, incubation of parasites with antisera specific for the large ectodomain of TgAMA1 reduces the frequency of invasion by ∼40% [15] and depletion of TgAMA1 expression using a conditional knockout strain virtually eliminates invasion [16]. *Plasmodium* AMA1 is a leading malaria vaccine candidate on the basis of several reports demonstrating that antisera targeting the ectodomain of *Plasmodium* AMA1 block erythrocyte invasion [19], [20], [21] and immunization with recombinant derivatives of *Plasmodium* AMA1 confer protection against the blood stage in animal models (reviewed in [22]).

Co-immunoprecipitation studies have led to the identification of TgRON2, TgRON4, TgRON5 and TgRON8 as members of the TgAMA1-associating MJ complex [8], [10], [12], [13]. Visualization of TgRON4/5/8 at the MJ has been confirmed [8], [10], [12], [13] but the subcellular localization of TgRON2 during invasion has been enigmatic. Despite biochemical evidence that is consistent with its localization to the MJ, the only visualization of TgRON2 outside of the rhoptry necks is as a secreted form that localizes to the tip of cytochalasin D-treated parasites (cytochalasin D acts to disrupt actin filaments, which are needed for parasite motility, and in this way blocks invasion but does not affect release of rhoptry proteins) [13]. Identification of *P. falciparum* AMA1-associating proteins demonstrates that the MJ complex composition is conserved, at least in part, in *P. falciparum* [9], [11], [23], with the notable exception that an orthologue of TgRON8 has not yet been identified in *Plasmodium spp*.

Characterization of the topology of the RONs during invasion and identification of the specific RON that binds AMA1 are crucial to gaining a better understanding of this unique complex. To this end, it has been reported that TgRON4/5/8 are secreted into the host cell where they localize to the cytosolic face of the host plasma membrane [12], [13]. TgRON2 has three predicted hydrophobic helices that have been postulated to span membranes [8], [10], [13], [24]. Combined with co-immunoprecipitation studies showing that TgRON2 is capable of independently associating with TgAMA1 [13] and TgRON4 [8], these results have led to the model that TgRON2 is the link between TgAMA1 on the parasite surface and the rest of the MJ complex, possibly spanning the host plasma membrane [12], [13].

To determine the role of TgRON2 in the function of the MJ, we generated transgenic parasites that could be used to visualize this protein at the MJ and used subdomains to better understand the TgRON2-specific interactions within the MJ complex. We demonstrate that a small, carboxy-terminal region of TgRON2 that spans what had been thought to be the third transmembrane domain associates with the ectodomain of TgAMA1 and that this interaction is critical for *T. gondii* invasion of, but not injection into, host cells.

## Results

### Visualization of TgRON2-HA at the moving junction by immunofluorescence

**Figure 1:**
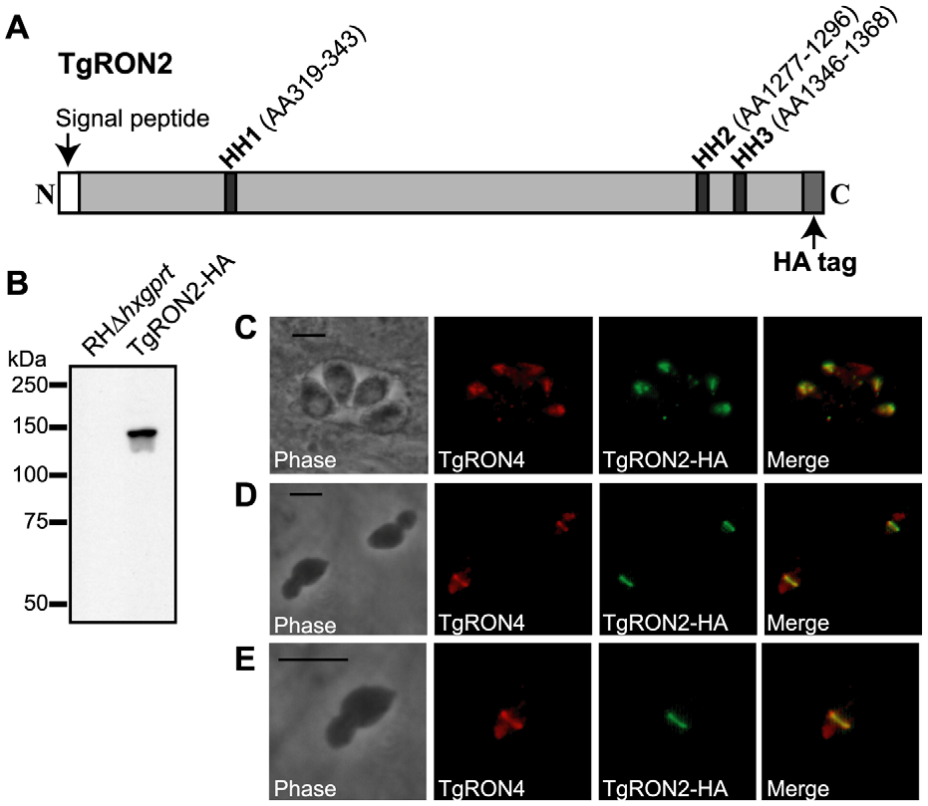
Visualization of TgRON2-HA in the rhoptry necks and at the MJ. (**A**) Schematic representation of the TgRON2 protein with the three putative hydrophobic helices (HH) designated by dark grey bars, with the spanning amino acids (AA) indicated above (AA positions are according to the TgRON2 sequence for the RH strain of *T. gondii*; Genbank accession number HQ110093). Parasites were engineered to endogenously express a derivative of TgRON2 fused to a C-terminal HA tag (TgRON2-HA). (**B**) Western blot analysis of the TgRON2-HA and parental parasites using the HA-specific rat monoclonal 3F10. (**C, D, E**) IFA analysis of infected HFF monolayers with intracellular (C) or partially-invaded (D, E) TgRON2-HA-expressing parasites. Infected monolayers were formaldehyde-fixed and permeabilized with methanol (C) or triton X-100 (D, E), then stained with rat anti-HA monoclonal 3F10 and rabbit anti-TgRON4 polyclonal sera [8]. The images shown in (E) are enlarged views of the parasite in the bottom, left side of (D). The scale bars represent 5 µm.

Initial attempts to localize TgRON2 within the MJ complex relied on antibodies to the native protein but were unsuccessful (data not shown; [13]). As an alternative strategy, we introduced an HA-tag to the C-terminus of TgRON2 in RHΔ*hxgprt* parasites by using a vector to homologously recombine a cassette that contains the coding sequence of the HA tag and the *HXGPRT* gene flanked by targeting sequences from upstream and downstream of the 3′ end of the *TgRON2* gene. Following selection for the HXGPRT marker using mycophenolic acid/xanthine, clones were identified and demonstrated by PCR to have the correct, homologously integrated sequences (data not shown). Examination of the resulting TgRON2-HA-expressing parasites by western blot using an HA-specific antibody demonstrated the expression of a single HA-tagged protein at the expected size of mature TgRON2 at ∼145 kDa (Figure 1B). Examination of the TgRON2-HA-expressing parasites by immunofluorescence (IFA) using an HA-specific antibody demonstrated that this fusion protein correctly localizes to the rhoptry necks of intracellular parasites as confirmed by co-localization with TgRON4 (Figure 1C).

To determine the localization of TgRON2 during invasion, TgRON2-HA-expressing parasites were used to infect human foreskin fibroblast (HFF) monolayers under conditions that synchronize invasion [25]. Parasites were permitted to invade for a short time (∼45 seconds) and then infected HFF monolayers were formaldehyde-fixed and permeabilized in triton X-100. Examination of TgRON2-HA parasites that were stalled in a partially invaded state using an HA-specific antibody demonstrated that TgRON2-HA can be visualized at the MJ, as determined by co-localization with the known MJ protein TgRON4 (Figure 1 D and E). These results provide direct confirmation that TgRON2 localizes to the MJ, thus confirming the biochemical data obtained previously that implicated TgRON2 in the MJ complex [8], [10], [12], [13].

### Two subregions of TgRON2 are able to independently associate with TgAMA1

**Figure 2:**
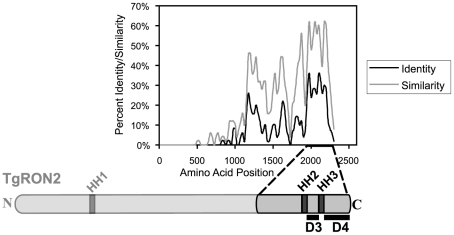
Alignment of TgRON2 with its orthologues reveals greatest sequence conservation in carboxy-terminal third of protein. The *Toxoplasma* RON2 polypeptide sequence was aligned with its orthologues in *Neospora caninum* and *Plasmodium spp.* (*P. falciparum* strain 3D7, *P. berghei*, *P. knowlesi*, *and P. vivax*) using ClustalX. The percent identity or percent similarity for 50 amino acid windows was calculated in 5 amino acid steps over the length of the alignment. The length of the alignment corresponds to the longest orthologue. Shown below is the relative region of TgRON2 that corresponds to the most conserved region of RON2 across the species. The regions between AA1293–1346 and AA1366–1479 were designated domain 3 (D3) and domain 4 (D4), respectively.

Like most of the other identified MJ components, TgRON2 orthologues are present within all Apicomplexa that show MJ formation during invasion, suggesting a conserved function for this protein. To identify regions of TgRON2 that are crucial for interacting with the other members of the MJ complex, therefore, we generated a multiple sequence alignment of TgRON2 and its orthologues in *Neospora caninum* and several *Plasmodium* species. We observed that the greatest sequence conservation among these orthologues is in the C-terminal-most third of the protein (Figure 2). To determine if this conserved region of TgRON2 is important for interactions with the remaining members of the MJ complex we generated protein fusions with glutathione S-transferase (GST) and used these in co-affinity purification studies. We chose to use regions outside of the hydrophobic helices as we anticipated that fusion proteins including these regions would not be soluble in *E. coli*, a prediction that was subsequently confirmed experimentally (data not shown). GST fusions with portions of TgRON2 N-terminal to the second putative hydrophobic helix (HH2), which spans residues 1277 to 1296, were also refractory to purification under soluble conditions (TgRON2 residue numbers are from Genbank accession HQ110093 with residue 1 as the start methionine); however, fusion of GST with TgRON2 amino acids 1293 to 1346 and 1366 to 1479, generating recombinant proteins GST-domain 3 (D3) and GST-domain 4 (D4), respectively, were successfully expressed and purified from *E. coli* (Figure 2 and data not shown).

**Figure 3:**
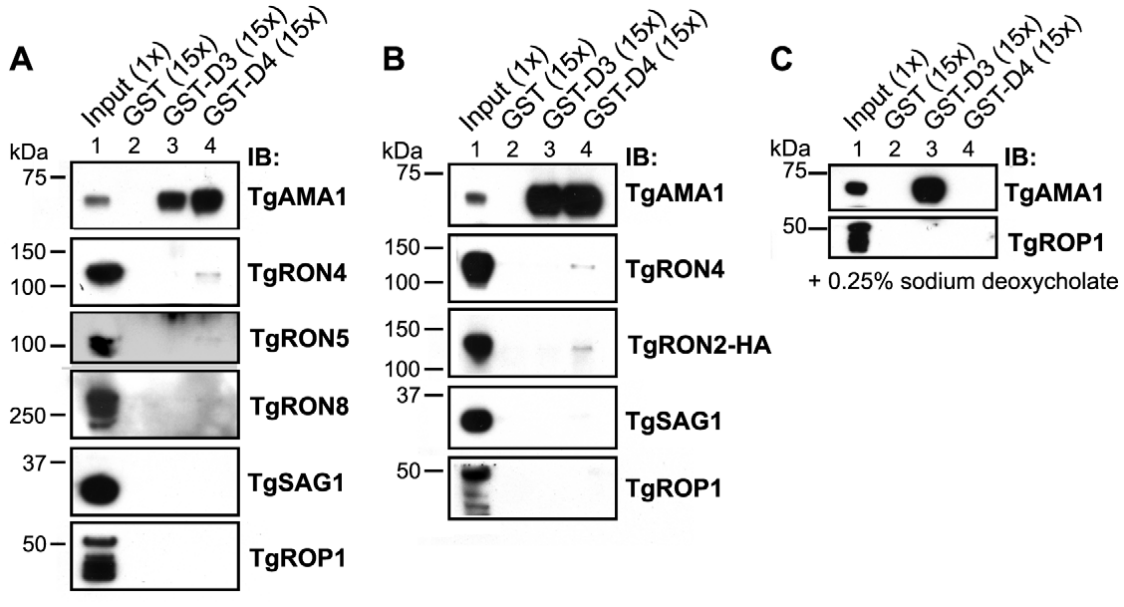
TgRON2 fusions GST-D3 and GST-D4 independently and specifically interact with TgAMA1 from parasite lysates. (**A**) Immunoblotting analysis of NP-40-solublized RHΔ*hxgprt* parasite lysates (Input) or material co-precipitated with molar equivalents of GST (lane 2), GST-D3 (lane 3) or GST-D4 (lane 4) was conducted to determine interaction with MJ complex members TgAMA1, TgRON4, TgRON5, and TgRON8. Western blots with each of the relevant antibodies (IB) are shown. TgSAG1 and TgROP1 serve as negative controls. Parentheses indicate loaded parasite equivalents. Size markers are indicated in kDa. (**B**) GST pull-down experiments were repeated as described in (A) using the TgRON2-HA parasites. (**C**) GST pull-down experiments were repeated as described in (A) but with supplementation of buffers with the ionic detergent sodium deoxycholate at a final concentration of 0.25% (w/v).

To determine if GST-D3 and GST-D4 are sufficient to co-purify any members of the MJ complex, molar equivalents of the GST fusion proteins as well as GST alone were bound to glutathione sepharose and then incubated with NP-40-soluble, RHΔ*hxgprt* parasite extracts in GST pull-down experiments. A sample of the input material and the co-purified, eluted material was then analyzed by immunoblotting using antisera for the different members of the MJ complex. Antisera to the abundant surface antigen, TgSAG1, or the abundant rhoptry protein, TgROP1, were also used to assess the specificity of the co-precipitations. We observed that both GST-D3 and GST-D4 but not GST alone efficiently co-precipitated TgAMA1 as determined by immunoblotting with the TgAMA1-specific monoclonal antibody B3.90 (Figure 3A, lanes 2–4). There was no detectable co-precipitation of the two negative controls (TgSAG1 and TgROP1), indicating that both the D3 and D4 domains of TgRON2 are independently capable of a specific interaction with TgAMA1.

Immunoblotting for TgRON4, TgRON5 and TgRON8 demonstrated that these proteins were not detected in the GST-D3 co-purified material (Figure 3A, lane 3) but a trace amount of at least TgRON4 and TgRON5 was consistently observed in the GST-D4 co-purified material (Figure 3A, lane 4). These results suggest that under these conditions GST-D4 may be able to interact with one portion of TgAMA1 without completely disrupting the native TgRON2:TgAMA1 interaction (via D3) such that a small amount of the entire complex is pulled down. As discussed further below, the D4 interaction may be too weak to hold the native complex together so that no native TgRON2 remains bound to the TgAMA1 in the GST-D3 co-precipitation.

To confirm that the associations between TgAMA1 and GST-D3 or GST-D4 were direct and not occurring via binding to a TgRON2:TgAMA1 complex, we repeated the GST pull-down experiments using the TgRON2-HA parasites followed by immunoblotting with an HA-specific antibody. The results show that, similar to TgRON4, TgRON2-HA was not detected in the material co-purifying with GST-D3 but trace amounts of it could be detected in the GST-D4 material (Figure 3B, lanes 3 and 4). Collectively, these results demonstrate that GST-D3 and GST-D4 are independently sufficient to affinity-purify TgAMA1 and that these associations are generally not as part of a complex with the other identified members of the MJ although GST-D4 may be able to associate with the complex without disrupting it completely.

To better characterize the associations between GST-D3/D4 and TgAMA1 we repeated the co-purification studies using more stringent conditions (i.e., in the presence of the ionic detergent sodium deoxycholate) that were shown to still yield intact MJ complexes in previous co-immunoprecipitation studies [8], [10]. Immunoblotting analysis of the co-purified material with the TgAMA1-specific antibody B3.90 demonstrate that while the association between GST-D3 and TgAMA1 was maintained, the association between GST-D4 and TgAMA1 is not seen under these conditions (Figure 3C, lanes 3 and 4). As sodium deoxycholate is considered to be more denaturing than NP-40 alone, these results suggest that the interaction between TgRON2 domain 3 and TgAMA1 is considerably stronger than the interaction involving domain 4.

### TgRON2 domains 3 and 4 associate with the ectodomain of TgAMA1

**Figure 4:**
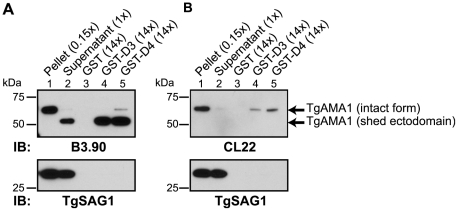
TgRON2 fusions GST-D3 and GST-D4 independently and specifically interact with the shed ectodomain of TgAMA1. Extracellular parasites were incubated under conditions to induce shedding of secreted TgAMA1 into culture supernatants. Immunoblotting analysis of pelleted parasites (lane 1), cleared culture supernatants (lane 2), or material from supernatants co-precipitated with molar equivalents of GST (lane 3), GST-D3 (lane 4) or GST-D4 (lane 5) was conducted using the monoclonal antibodies B3.90 (A), specific for the TgAMA1 N-terminus, or CL22 (B), specific for the TgAMA1 C-terminus. The former detects the intact, integral membrane form (∼65 kDa) and the shed ectodomain (∼53 kDa) while CL22 detects only the intact form. TgSAG1 serves as a negative control. Parentheses indicate loaded parasite equivalents. Size markers are indicated in kDa.

Current models of the assembly of the MJ complex predict that TgRON2 is associated with the host cell where it acts as a receptor for the ectodomain of TgAMA1 [12], [13]. To test this hypothesis and determine whether D3 and/or D4 are the domains of TgRON2 that are functioning in this association we used GST-D3 and GST-D4 in GST pull-down experiments with parasite culture supernatants containing the shed, N-terminal ectodomain of TgAMA1 [15], [26]. To discriminate between the shed and the intact, full-length forms of TgAMA1, immunoblotting was performed using monoclonal antibodies specific for either the C-terminal intracellular domain of TgAMA1 (CL22; [15]) or the N-terminal extracellular domain (B3.90; [26]). The results showed that both GST-D3 and GST-D4 but not GST alone efficiently co-precipitate the more rapidly migrating (shed) form of TgAMA1 (Figure 4A). The identity of this as the shed form was confirmed by its failure to react to CL22 (Figure 4B). A trace amount of contaminating, intact, full-length TgAMA1 in the supernatant material was also co-precipitated, as expected, since this also includes the entire ectodomain (Figure 4 A and B, lanes 4 and 5). The specificity of these co-purification studies was confirmed by a complete lack of enrichment for the abundant surface antigen TgSAG1 (Figure 4). These results demonstrate that both GST-D3 and GST-D4 independently and specifically interact with the ectodomain of TgAMA1.

**Figure 5:**
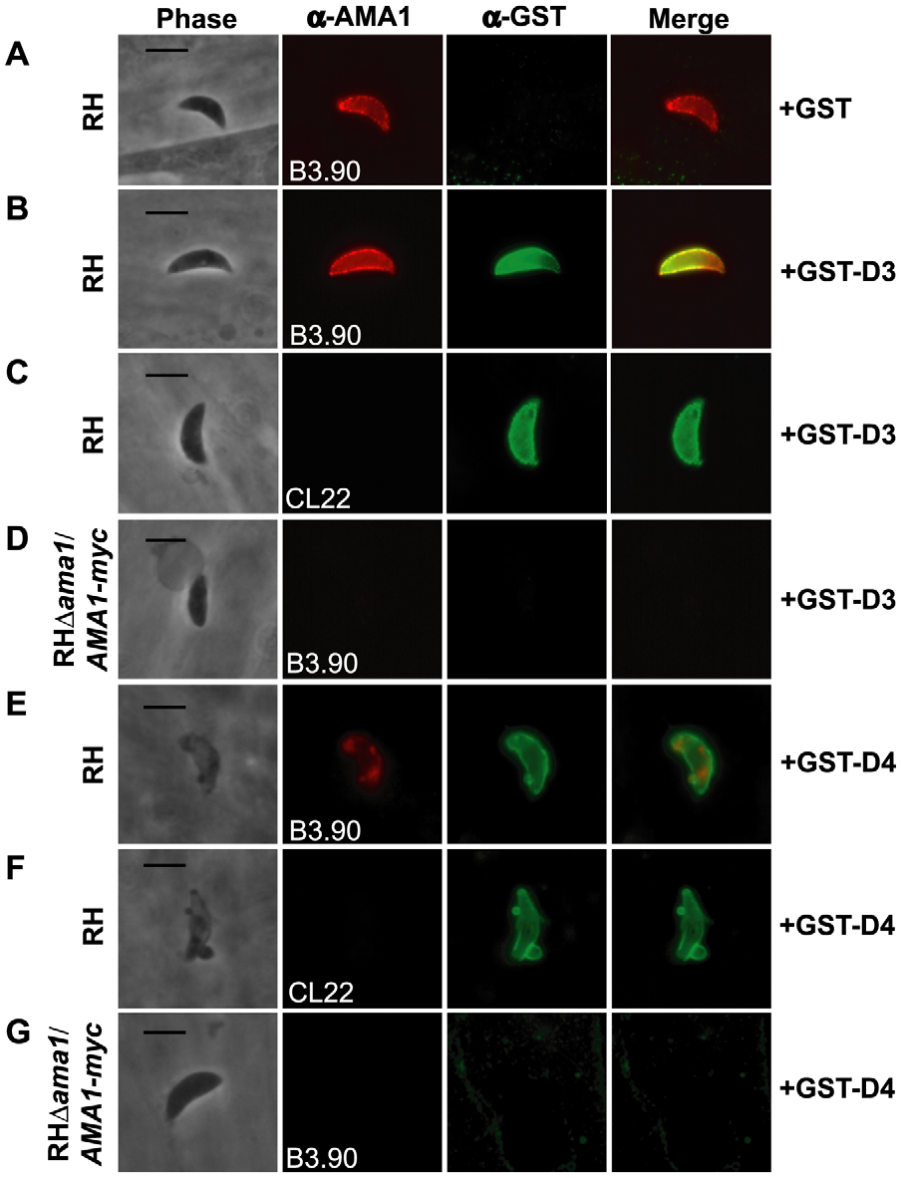
TgRON2 fusions GST-D3 and GST-D4 bind to TgAMA1 on the surface of parasites. Extracellular RH (A, B, C, E, and F) or RHΔ*ama1*/*AMA1-myc* (D and G) parasites were pre-treated with, and then permitted to infect HFF monolayers in the presence of, molar equivalents of GST alone (A), GST-D3 (B–D), or GST-D4 (E–G). Infected monolayers were formaldehyde-fixed and stained in the absence of permeabilization using the TgAMA1 monoclonal B3.90 (A, B, D, E, and G, panel 2), specific for the ectodomain, or the TgAMA1 monoclonal CL22 (C and F, panel 2), specific for an epitope inside the parasite cytosol, or rabbit antisera specific for GST (A–G, panel 3). Merged images for the anti-TgAMA1 and anti-GST antibodies are shown in panel 4 for each set. The RHΔ*ama1*/*AMA1-myc* parasites were grown under conditions known to deplete AMA1-myc expression [16]. Scale bars represent 5 µm.

To determine whether GST-D3 and GST-D4 can interact with the ectodomain of TgAMA1 on intact RH parasites, we used IFA and anti-GST antibodies. Freshly prepared, extracellular parasites were pre-incubated with molar equivalents of GST or GST-D3 (see below for details on GST-D4) then permitted to settle on HFF monolayers under conditions that were not permissive for invasion, followed by a brief shift to invasion-permissive conditions. Infected monolayers were fixed and then stained in the absence of any added permeabilizing agents using a GST-specific antibody and the TgAMA1 ectodomain-specific monoclonal antibody B3.90. The results showed that parasites pre-incubated with GST-D3, but not GST alone, exhibit surface staining with the GST-specific antibody (Figure 5 A and B). As a control to confirm that the parasite plasma membrane was not permeabilized in the fixation process we also stained parasites with the monoclonal antibody CL22, which is specific for the intracellular C-terminus of TgAMA1 (Figure 5C). No staining with CL22 was observed, confirming the intactness of the membranes. These results indicate an association between GST-D3 and the exposed, ectodomain of TgAMA1, which under these conditions is distributed over the entire surface of the parasite (Figure 5 A and B, second panel; [15], [26].

To verify that binding of GST-D3 to the parasite surface is via TgAMA1, we used the conditional TgAMA1 knockout strain (RHΔ*ama1*/*AMA1-myc*) [16] grown in the presence of anhydrotetracycline (Atc), which turns off TgAMA1 expression. Freshly prepared, extracellular RHΔ*ama1*/*AMA1-myc* parasites grown under such conditions were incubated in the presence of GST-D3 and stained, as described above. Staining for TgAMA1 using the monoclonal B3.90 confirmed that these parasites were not expressing detectable levels of TgAMA1 (Figure 5D, second panel) and that, in contrast to parasites that express wild-type levels of TgAMA1, there was no detectable binding of GST-D3 (Figure 5D, third panel). These results demonstrate that the surface staining observed with GST-D3 is dependent on TgAMA1.

Similar analyses were conducted with GST-D4. By IFA, we observed that multiple, independent preparations of GST-D4 stain the surface of RH parasites, as predicted, but these preparations were toxic, causing what appears to be membrane blebs and, perhaps as a result, alteration of TgAMA1 staining in more than 50% of the parasites treated (Figure 5 E and F). In contrast, there was no detectable binding of GST-D4 on most (>80%) of RHΔ*ama1*/*AMA1-myc* parasites that were grown in the presence of Atc (Figure 5G). However, we did observe a toxic effect, as well as GST staining, in less than 20% of the RHΔ*ama1*/*AMA1-myc* parasites (data not shown). While there was no detectable TgAMA1 on these parasites as determined by B3.90 staining (data not shown), it is possible that residual surface-localized TgAMA1, while below the limit of detection, may have been sufficient for binding by GST-D4. Collectively, these results indicate that binding of GST-D4 to the surface of *Toxoplasma* occurs in an AMA1-specific manner but this binding elicits a morphological change in the parasite plasma membrane that precluded further functional analyses.

### Pre-incubation of parasites with GST-D3 decreases host cell invasion

**Figure 6:**
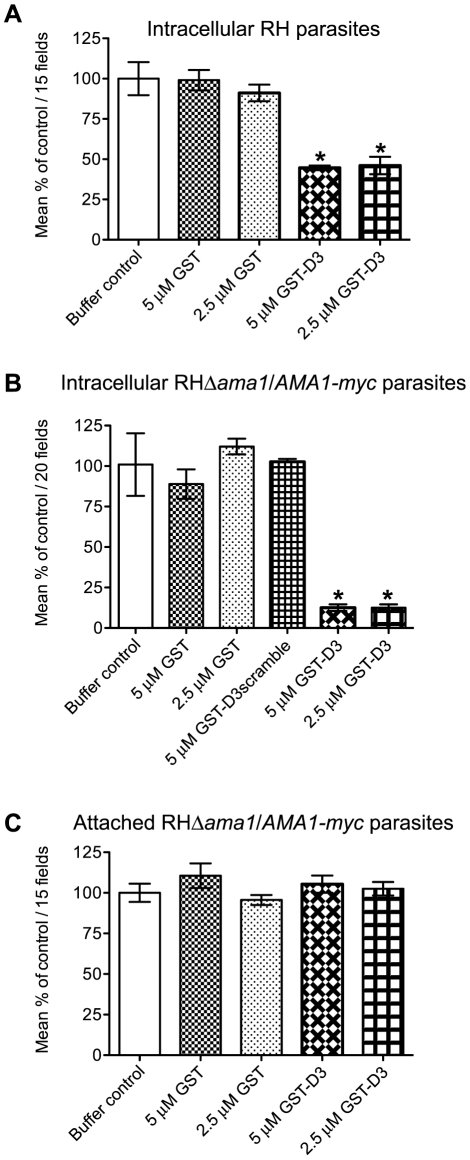
Pre-incubation of RH and RHΔ*ama1*/*AMA1-myc* parasites with GST-D3 decreases invasion efficiency. (**A, B**) Extracellular RH (A) or RHΔ*ama1*/*AMA1-myc* (B) parasites were pre-treated with a buffer control or indicated molar equivalents of GST alone, GST-D3, or GST-D3scramble (B only) and then permitted to infect HFF monolayers for 15 minutes using temperature-based synchronized invasion conditions. The number of intracellular parasites was determined by differential staining of the extracellular vs. total parasites before and after detergent permeabilization. The number of intracellular parasites was determined for 15 (A) or 20 (B) randomly-selected fields from three coverslips for each condition tested. The invasion levels for each condition are shown relative to the buffer-treated control (shown are means with standard deviation). An asterisk indicates a statistically significant reduction in invasion relative to the GST controls (unpaired Student's *t*-test), with *p*<0.0099 (A) or *p*<0.0002 (B). (**C**) To determine if GST-D3 treatment affects attachment, RHΔ*ama1*/*AMA1-myc* parasites were pre-treated with molar equivalents of GST alone, GST-D3, or a buffer control as described above and then permitted to attach to formaldehyde-fixed HFF monolayers. The number of stained, attached parasites was counted in 15 randomly-selected fields from three coverslips for each condition tested. The attachment levels for each condition are shown relative to the buffer-treated control (shown are means with standard deviation).

Given that GST-D3 associates with the ectodomain of TgAMA1 on the surface of intact parasites, we predicted that pre-incubation of extracellular parasites with GST-D3 would result in an invasion-inhibitory phenotype. To test this hypothesis, equivalent numbers of freshly prepared, extracellular RH parasites were pre-incubated in medium supplemented with molar equivalents of GST-D3, GST alone, or a buffer control and then allowed to invade host cells using a temperature-shift assay to synchronize the process. Following ∼15 minutes at an invasion-permissive temperature, infected monolayers were fixed and analyzed by IFA. Extracellular vs. intracellular parasites were identified by sequential staining for TgSAG1, before and after detergent-permeabilization of the host cells. While treatment of RH parasites with GST alone did not significantly alter the number of intracellular parasites compared to the buffer-treated control parasites, treatment with GST-D3 resulted in a dose-dependent decrease of up to ∼55% (Figure 6A and Supplemental Figure S1A). This level of inhibition is similar to that previously reported with anti-TgAMA1 antibodies [15] and is consistent with disruption of TgAMA1 function through binding of GST-D3.

Previous studies demonstrated that the levels of TgAMA1 in wild-type parasites are apparently present in excess: RHΔ*ama1*/*AMA1-myc* parasites that, even in the absence of Atc, only express ∼10% of the wild-type levels of TgAMA1 are fully competent for invasion [16]. Given this, we postulated that the excess TgAMA1 on the surface of RH parasites might act to absorb GST-D3 thus decreasing the effect of this protein on the biologically-relevant minority of TgAMA1 actually involved in invasion. To test this hypothesis and to confirm that the invasion-inhibitory phenotype we observe with GST-D3 treatment of RH parasites is dependent upon TgAMA1, we repeated the invasion assays using the RHΔ*ama1*/*AMA1-myc* parasites. As seen with the RH parasites, pre-incubation of RHΔ*ama1*/*AMA1-myc* parasites with GST alone did not affect the number of intracellular parasites, as compared to the buffer control (Figure 6B). Strikingly, however, we observed that pre-incubation of the RHΔ*ama1*/*AMA1-myc* parasites with GST-D3 resulted in a dose-dependent decrease of up to ∼87% in invasion efficiency (Figure 6B and Supplemental Figure S1B). To confirm the specificity of this effect, RHΔ*ama1*/*AMA1-myc* parasites were also incubated with a GST protein fused to a scrambled version of the D3 peptide sequence (designated GST-D3scramble), which does not affinity-purify TgAMA1 from parasite extracts or culture supernatants (data not shown). The percentage of intracellular parasites following treatment with GST-D3scramble was similar to that seen for parasites incubated with the buffer or GST alone controls (Figure 6B) demonstrating that the invasion-inhibitory phenotype observed with GST-D3 is indeed highly specific. A specific interaction with TgAMA1 is argued by the fact that the effect of GST-D3 treatment was much more pronounced for the RHΔ*ama1*/*AMA1-myc* parasites than it was for wild-type.

To determine if treatment of parasites with GST-D3 affects host cell attachment we tested the ability of the RHΔ*ama1*/*AMA1-myc* parasites treated with GST alone or GST-D3 to attach to formaldehyde-fixed HFF monolayers. *T. gondii* will attach to fixed monolayers but cannot invade thus permitting us to test attachment independently of invasion [27]. Using this assay we did not observe any statistically significant difference between the number of attached parasites following treatment with GST alone, GST-D3, or the buffer control (Figure 6C). To determine if treatment of parasites with GST-D3 affects gliding motility, we examined the number and type of “gliding” trails deposited on glass coverslips by RHΔ*ama1*/*AMA1-myc* parasites treated with GST-D3 or GST alone. Again, we saw no detectable difference in the number or form of such trails following either treatment (data not shown). Collectively, these results demonstrate that GST-D3 impedes invasion of host cells, rather than attachment or gliding motility.

**Figure 7:**
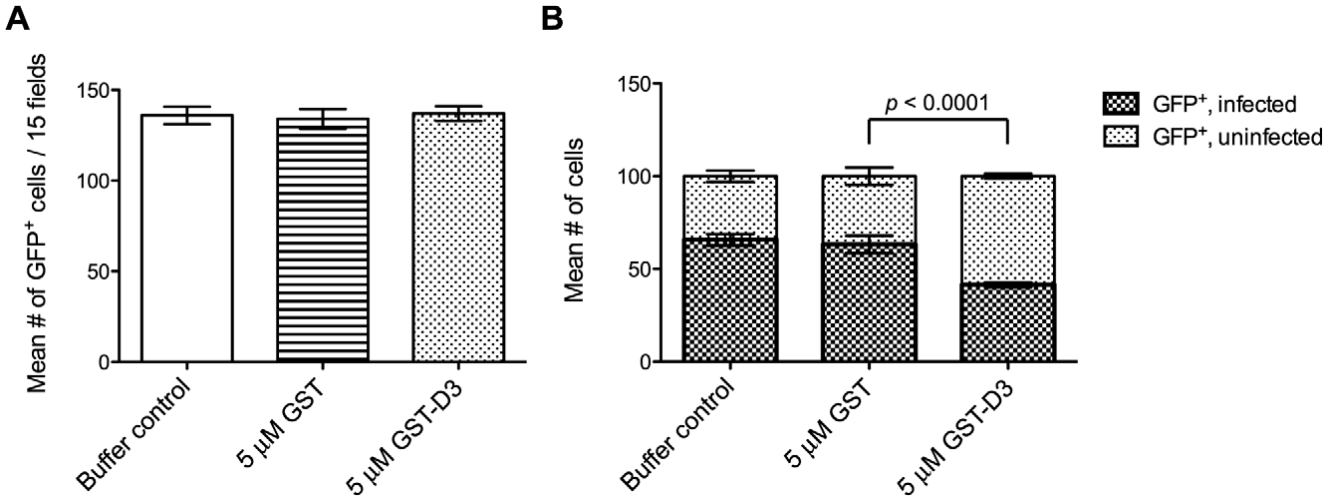
Pre-incubation of parasites with GST-D3 does not affect rhoptry bulb secretion. Extracellular SeCreEt parasites were analyzed for their ability to inject the rhoptry fusion protein toxofilin-Cre into Cre-reporter host cells following treatment with a buffer control, GST, or GST-D3. (**A**) The number of GFP-positive cells was determined in 15 randomly selected fields from four coverslips for each condition tested (shown are means with standard deviation). (**B**) The ratio of uninfected to infected GFP-positive cells, counting a total of 100 cells, was determined in randomly selected fields from four coverslips for each condition tested (shown are means with standard deviation).

During or soon after invasion, *Toxoplasma* tachyzoites inject soluble rhoptry bulb proteins (“ROPs”) into the host cell as a means to co-opt host cell functions [28], [29], [30]. To determine if treatment with GST-D3 affects the injection of ROPs, we analyzed “SeCreEt” parasites that have been engineered to inject a protein consisting of the soluble ROP, toxofilin, fused to Cre recombinase [31]. Upon invasion of a Cre-reporter host cell by a single SeCreEt parasite, Cre-mediated recombination results in host cell expression of GFP. It should be noted that under normal conditions, Cre-mediated recombination can be observed in occasional uninfected cells; these uninfected, GFP-positive cells are thought to result from abortive invasion events or cells that divide following invasion by the parasites [31]. To determine if GST-D3 treatment affects injection of the toxofilin-Cre fusion, equivalent numbers of freshly prepared, extracellular SeCreEt parasites were pre-incubated in medium supplemented with molar equivalents of GST-D3, GST alone, or a buffer control and then allowed to invade Cre-reporter host cells using a temperature-shift assay to synchronize the process. Following ∼30 minutes at an invasion-permissive temperature, infected monolayers were washed three times and then incubated in media supplemented with GST-D3, GST alone, or a buffer control for an additional ∼24 hours. Infected monolayers were then analyzed for the number of GFP-positive host cells. Using this assay, we saw no significant difference in the total number of GFP-positive host cells following treatment with GST-D3, GST, or a buffer control (Figure 7A). While the total number of GFP-positive cells did not differ, the percentage of the GFP-positive cells that were infected with SeCreEt parasites was significantly reduced following treatment with GST-D3, as expected (Figure 7B). The extent of this reduction in invasion efficiency was less than that observed in the experiments reported in figure 6, likely as a result of the much longer time needed for the Cre-reporter assay (∼24 hours vs. ∼15 minutes); the additional time may allow some number of the GST-D3-inhibited parasites to eventually enter. Regardless, these results clearly demonstrate that while GST-D3 binding to AMA1 can block invasion of host cells, it has little if any effect on the injection of the rhoptry bulb contents.

Due to its toxic effect on the parasites, similar analyses could not be attempted with GST-D4. It is unclear if this effect on the parasites is biologically relevant but, regardless, it precluded attempts to determine if preincubation with GST-D4 has an effect on *T. gondii* invasion.

## Discussion

The results presented here demonstrate that TgRON2 localizes to the MJ of invading parasites and that at least two regions within the last ∼200 amino acids of TgRON2 independently and specifically associate with the ectodomain of TgAMA1. A short sub-region of just 54 amino acids (D3) was found to block invasion, also in a TgAMA1-dependent manner. The invasion-inhibitory effect of this interaction is unlikely to be due simply to the non-specific presence of GST-D3 on the surface as coating *Toxoplasma* with antibodies to the abundant surface antigen TgSAG1 does not impair host cell attachment and invasion [27], [32]. Instead, our results strongly argue for a critical role for the association of TgAMA1 and TgRON2 in productive invasion of host cells.

The D3 and D4 regions of TgRON2 identified as interacting with TgAMA1 are separated by 19 amino acids that were previously predicted to span a membrane [8], [10], [13], [24]. As both regions interact with the ectodomain of TgAMA1, however, our data clearly indicate that the entire C-terminus of TgRON2, encompassing D3, HH3, and D4, is on the extra-cytosolic region of the MJ during invasion; i.e. HH3 does not span a membrane. We note that both HH2 and HH3 exhibit a high degree of identity between TgRON2 and its orthologues in other Apicomplexa, thus indicating a key role for these regions beyond simple hydrophobicity. Given the conserved nature of the hydrophobic cleft in AMA1 from *Toxoplasma* and *Plasmodium* and the importance of this region in PfAMA1 binding to the MJ complex [11], [14], [33], our results suggest the possibility that HH3, along with D3 and D4, specifically associates with the TgAMA1 face that includes the hydrophobic cleft via hydrophobic∶hydrophobic interactions. These results also provide a likely molecular basis for recent reports using *P. falciparum* where the invasion-inhibitory R1 peptide and anti-PfAMA1 antibody 4G2 were shown to inhibit invasion [11], [14]; R1 has substantial similarity to a portion of PfRON2 overlapping HH3 and the N-terminal end of domain 4 (V**FA**EFL**P**-**LF** SKF-**G**SRM-H**ILK** for the R1 peptide vs. L**FA**SIG**P**Y**LF** APMA**G**LAVWN**ILK** for PfRON2 with similar residues underlined and identical residues underlined and in bold). Analysis of the association between the C-terminus of RON2 and the ectodomain of AMA1 by co-crystallization will be needed to resolve the exact nature of their association.

While this manuscript was in preparation, we learned of similar results by others [34]. Their data corroborate ours and also show that the N-terminus of TgRON2 is inside the host cell. Combined with the results presented here showing that the D3 and D4 domains are both outside the host cell, it would appear that TgRON2 spans the host plasma membrane an odd number of times. Given that HH3 is apparently not a TM domain, it is most likely that only one of HH1 and HH2 can be a true trans-membrane domain although we cannot exclude the possibility that this topology is accomplished through the existence of a cryptic, third such span. The fact that TgRON2 and TgRON4, which is reported to be inside the host cell plasma membrane [13], also appear to form a tight interaction with one-another [8] supports a model wherein TgRON2 spans the host membrane with D3/D4 binding to TgAMA1 on the parasite surface and with some other portion of TgRON2, N-terminal of D3, binding TgRON4 inside the host cell.

While it seems simplest to explain the invasion-inhibitory effect of GST-D3 as being due to a steric block in the binding of TgRON2 to TgAMA1, we cannot exclude the possibility that the effect is also a result of interference with a key signaling event. GST-D3-binding did not detectably affect the ability of the parasites to inject rhoptry bulb proteins. This suggests that rhoptry neck proteins (e.g., TgRON2) and at least some amount of rhoptry bulb proteins are injected at the earliest stages of invasion. This is not unexpected based on published data showing injection of ROP9 by a parasite that is less than a third of the way into the host cell [28]. The fact that the RONs are presumably injected before MJ formation (since they must gather into a complex inside the host cells as part of MJ assembly) and the fact that there is no known separation of the bulb and neck compartments also makes the failure of GST-D3 to block ROP injection not unexpected. Hence, it appears that rhoptry bulb and neck release are not separable phenomena, although by virtue of their physical proximity to the apical end of the rhoptries, the RONs are likely injected before the ROPs.

Overall, and regardless of whether the GST-D3-mediated block in MJ formation is steric or through aberrant signaling, our data clearly indicate that the TgAMA1/TgRON2 interaction is key for invasion. It is reasonable to expect, therefore, that synthetic compounds that block this interaction will be potent anti-parasitic agents. Similarly, the D3–D4 region of RON2 should receive serious consideration as a possibly synergistic addition to current AMA1-based vaccines.

## Materials and Methods

### Host cell culture and parasites

Human foreskin fibroblasts (HFFs) were cultured in complete Dulbecco's modified Eagle's medium (DMEM; Invitrogen, Carlsbad, CA) supplemented with 10% heat-inactivated fetal calf serum (Hyclone, Logan, UT), 2 mM L-glutamine, 100 U ml^−1^ penicillin and 100 µg ml^−1^ streptomycin.

*Toxoplasma gondii* (RH strain) lacking a functional hypoxanthine-xanthine-guanine phosphoribosyltransferase (*HXGPRT*) gene (designated RHΔ*hxgprt*) [35] were cultured by serial passage on confluent monolayers of HFFs in complete DMEM at 37°C with 5% CO_2_. Selection and maintenance of transfected parasites expressing the *HXGPRT* cassette were cultured in complete DMEM supplemented with 50 µg/ml of mycophenolic acid and 50 µg/ml of xanthine (MPA/XAN).

### Generation of TgRON2-HA parasites

All primers used in these studies are listed in Table S1.

To generate a *Toxoplasma* strain where the endogenous *TgRON2* locus was replaced with a copy of *TgRON2* fused to a C-terminal hemagglutinin (HA) epitope tag, a plasmid with ∼2.5–3 kb of 5′ and 3′ homologous *TgRON2*-targeting sequences, flanking the HA tag coding sequence and the *HXGPRT* gene, was constructed. The 3′ targeting sequence, including the *TgRON2* 3′UTR, was PCR amplified from *Toxoplasma* genomic DNA (RH strain) using primers A and B, and then introduced into the p*TKO* vector (kindly provided by G. Zeiner, Stanford University, Stanford, CA) using the NheI and ApaI sites. The 5′ targeting sequence encompassing the last ∼3 kb of *TgRON2*, but excluding the native stop codon, was PCR amplified from *Toxoplasma* genomic DNA using primers C and D. The resultant PCR product, which also contained the coding sequence for the HA tag and a downstream stop codon in frame with the last codon of *TgRON2*, was introduced into the derivative of *pTKO* that contained the 3′ targeting sequence using the KpnI and EcoRV sites, to generate the complete *pT*-*TgRON2-HA* tagging vector.

The *pT*-*TgRON2-HA* vector was introduced into RHΔ*hxgprt* parasites by electroporation as described previously [36], selected for by passage in complete DMEM supplemented with MPA/XAN, and then individual *Toxoplasma* clones were isolated by serial dilution. Insertion of the HA tag and the *HXGPRT* gene into the *TgRON2* locus was confirmed by PCR using two primers sets; each set included one primer that hybridized to the *Toxoplasma* genome outside of the targeting regions cloned into the *pT*-*TgRON2-HA* and one primer that hybridized to a unique sequence present in the targeting vector that was retained in the *Toxoplasma* genome after the double-recombination event. Proper integration at the 5′ or 3′ ends was confirmed using primers E and F or G and H, respectively.

### Generation of GST-TgRON2 fusion proteins

To generate TgRON2 fusion proteins with a N-terminal GST tag, subregions of the *TgRON2* cDNA were PCR-amplified from a *Toxoplasma* RH strain cDNA library and introduced into *pGEX-6P1* (Agilent) using the BamHI and EcoRI sites. To generate GST-D3, the coding sequence for TgRON2 amino acids 1293–1346 were PCR amplified using primers I and J. To generate GST-D4, the coding sequence for TgRON2 amino acids 1366–1479 was PCR amplified using primers K and L. An unbiased scrambled derivative of D3 was generated computationally with the RandSeq tool (ExPASy proteomics Server, Swiss Institute of Bioinformatics) and the resulting amino acid sequence, designated D3scramble, was reverse translated to a coding sequence for the purpose of primer design. The primers used to generate GST-D3scramble (primers M and N) were designed with 16 base-pairs of overlapping sequence so that they served both as primers and template in a PCR reaction. The amplified product was inserted into *pGEX-6P1* as described above.

Production and purification of the GST proteins from *E. coli* strain Rosetta (Novagen) were done essentially as described previously [37]. Concentrated, purified proteins were stored at −80°C in buffer containing 10 mM Tris-HCl pH 8.0, 150 mM NaCl, and 10% glycerol.

### GST pull-down experiments

To identify *Toxoplasma* proteins that interact with GST-D3/D4, approximately 4×10^8^ extracellular RHΔ*hxgprt* or TgRON2-HA parasites were washed three times in 1× phosphate-buffered saline (PBS) and then lysed on ice in 1 ml of lysis buffer (10 mM Tris-HCl pH 8.0, 150 mM NaCl, 1 mM EDTA, 0.1% NP-40) supplemented with Complete EDTA-free protease inhibitors (Roche). The cleared, NP-40-solublized lysate was divided equally into three tubes and each fraction was supplemented with 4B Glutathione-sepharose beads (GE Healthsciences) that were pre-bound with 0.5 µM of GST, GST-D3, or GST-D4. The lysate suspensions were rotated at room temperature for approximately two hours and the bound beads were then washed three times in lysis buffer, followed by elution of the GST fusion proteins and any co-purified parasite proteins by boiling for ∼5 minutes in 2× SDS sample buffer (125 mM Tris-HCl pH 7.0, 4% SDS, 20% glycerol, 0.005% bromophenol blue) supplemented with 10% β-mercaptoethanol. Lysate samples and eluted material were separated on 4–12% gradient Bis-Tris gels (Invitrogen) and analyzed by western blot using the appropriate antibodies.

To better understand the nature of the interaction between TgAMA1 and GST-D3 or GST-D4, the GST pull-down experiments were conducted as described above in buffers supplemented with sodium deoxycholate at a final concentration of 0.25% (w/v).

To determine if the GST fusion proteins bind to the shed ectodomain of TgAMA1, freshly extracellular RHΔ*hxgprt* parasites were pelleted and washed three times in Hanks Buffered Saline Solution (HBSS) supplemented with 20 mM HEPES pH 7.4. Washed parasites were passed through a 5 µm filter, pelleted, and then resuspended at a density of ∼3.5×10^8^ parasites per ml in serum-free DMEM supplemented with 20 mM HEPES pH 7.4 and 2 µM ionomycin (Sigma). Following incubation at 37°C for approximately 30 minutes, the parasites were pelleted. The resulting pellet was washed twice in PBS and then resuspended in one ml of SDS sample buffer (without reducing agents) and saved as the “pellet” fraction while the resulting supernatant was spun again at 100,000×g for 45 minutes at 4°C. A sample of the cleared “supernatant” fraction was saved and the remainder was supplemented with NaCl (150 mM final) and NP-40 (0.1% (v/v) final), then equally divided into three tubes followed by incubation with 0.5 µM GST, GST-D3, or GST-D4 proteins pre-bound to glutathione sepharose. The GST pull-down experiments were conducted as described above. Elution of the GST fusion proteins and any co-purified parasite proteins was by boiling for ∼5 minutes in SDS sample buffer without any reducing agents.

### Western blot analyses

To detect TgRON2-HA by western blot, intracellular RHΔ*hxgprt* or TgRON2-HA-expressing parasites were released from infected HFFs by scraping and passage through a 27 gauge needle. Released parasites were washed twice in 1× PBS and then counted. Lysates were generated for each strain by pelleting 10^6^ parasites and resuspending each pellet in 15 microliters of 2× SDS sample buffer supplemented with 10% β-mercaptoethanol. Following boiling for 5 minutes, 10 microliters of each lysate were separated by SDS-PAGE and TgRON2-HA was detected using the anti-HA rat monoclonal antibody 3F10 conjugated to horseradish peroxidase (HRP) (Roche).

In western blot analyses of the GST pull-down material, TgRON4/8 were detected using rabbit or mouse polyclonal antisera, respectively [8], [12]. TgRON5 was detected using rabbit polyclonal antisera specific for the N-terminus of the protein (kindly provided by the Bradley laboratory, University of California-Los Angeles, Los Angeles, CA). TgAMA1 was detected with mouse monoclonals B3.90 [26] or CL22 [15]. TgSAG1 was detected using rabbit polyclonal sera (a gift from M. Grigg, NIH). TgROP1 was detected using the mouse monoclonal antibody Tg49 [38]. All goat anti-mouse and anti-rabbit secondary antibodies were HRP-conjugated (Biorad).

### Immunofluorescence assays

All secondary AlexaFluor-conjugated antibodies were obtained from Molecular Probes.

To visualize the TgRON2-HA fusion protein in the rhoptry necks, HFF monolayers infected with TgRON2-HA-expressing parasites were fixed in 1× PBS containing 2.5% formaldehyde (EM Biosciences) ∼16 hours post-infection. These fixed HFF monolayers were then permeabilized using 100% methanol and stained with the HA-specific rat monoclonal 3F10 (Roche) and rabbit anti-RON4 polyclonal sera followed by AlexaFluor488-goat-anti-rat antibody and AlexaFluor594-goat-anti-rabbit antibody, respectively. Coverslips were mounted onto glass slides using Vectashield (Vector Laboratories) and then examined using 100× oil-immersion lens on an Olympus BX60 upright fluorescent microscope. All digital images were obtained using Image-Pro Plus and the same exposure parameters were used for all comparison sets.

To visualize TgRON2-HA at the MJ of partially invaded parasites, extracellular parasites were prepared for synchronous invasion of HFF monolayers using high potassium buffers essentially as described [25]. Approximately 45 seconds following the addition of invasion-permissive media, infected monolayers were fixed in 1× PBS containing 2.5% formaldehyde, permeabilized with 1× PBS containing 0.2% triton X-100 and then the HA-tagged proteins and TgRON4 were detected as described above.

To visualize binding of GST proteins to the surface of intact parasites, ∼10^8^ freshly extracellular (by natural egress) RH or RHΔ*ama1*/*AMA1-myc* parasites (grown for 48 hours in the presence of anhydrotetracycline (Atc) as described [16]) were washed twice in DMEM with 2% FBS and then passed through a 5 µm filter. Parasites were incubated in DMEM containing 2% FBS that was supplemented with either 5 µM GST, 5 µM GST-D3, or 5 µM GST-D4 by rotation at 37°C for ∼20 minutes. Parasites were then chilled in an ice water bath to prepare them for temperature-based synchronized invasion. This chilled *Toxoplasma* suspension was then added to pre-chilled HFF monolayers grown on glass coverslips. Parasites were permitted to settle onto the HFFs in an ice water bath for ∼12 minutes prior to invasion. To initiate invasion, the plate was then transferred to a 37°C water bath for ∼1 minute. Infected monolayers were washed twice in 1× PBS and then fixed in 1× PBS containing 2.5% formaldehyde. Unless otherwise stated, all staining steps were conducted in 1× PBS supplemented with 3% bovine serum albumin (BSA; Sigma) in the absence of added permeabilization agents. TgAMA1 was visualized using the mouse monoclonal antibodies B3.90 or CL22 and AlexaFluor594-goat-anti-mouse. GST proteins were visualized using rabbit anti-GST antisera (ICL) and AlexaFluor488-goat-anti-rabbit.

### Invasion assay

The preparation of *Toxoplasma* for the invasion assays was conducted essentially as described above for the IFA analysis of GST-treated parasites with minor modifications. Briefly, washed RH or RHΔ*ama1*/*AMA1-myc* parasites were incubated in DMEM with 2% FBS supplemented with 2.5–5 µM GST, 2.5–5 µM GST-D3, 5 µM GST-D3scramble (RHΔ*ama1*/*AMA1-myc* parasites only) or 1.5% volume of a buffer control (10 mM Tris-HCl pH 8.0, 150 mM NaCl, 10% glycerol). Using temperature-based synchronization as described above, invasion was permitted for approximately 15 minutes at 37°C, and then infected HFF monolayers were washed gently once in 1× PBS and fixed in 1× PBS containing 2.5% formaldehyde. To stain only the extracellular parasites, fixed monolayers were stained with rabbit anti-SAG1 antiserum followed by AlexaFluor594-goat-anti-rabbit. To stain all parasites, the infected monolayers were then permeabilized with 1× PBS containing 0.2% triton X-100 followed by staining with the anti-SAG1 mouse monoclonal antibody DG52 [39] and AlexaFluor488-goat-anti-mouse. The numbers of green (intracellular and extracellular) and red (extracellular) *Toxoplasma* were counted in 15 (RH) or 20 (RHΔ*ama1*/*AMA1-myc*) randomly selected fields on each of three separately mounted coverslips for each condition and visualization was performed using a 20× lens on a Nikon Eclipse TE300 microscope. All digital images were obtained using Image-Pro Plus and parasites were quantified using ImageJ. Results are representative of data from at least three independent experiments.

### Attachment assay

Extracellular RHΔ*ama1*/*AMA1-myc* parasites were prepared and treated with the GST proteins as described above. Following treatment with the GST proteins, the parasite suspensions were added directly to HFF monolayers on coverslips that had been formaldehyde-fixed prior to the addition of *Toxoplasma*, essentially as described [27] and permitted to settle for 15 minutes at 37°C. Monolayers of formaldehyde-fixed HFFs with attached parasites were washed once (for consistency with the invasion assays) and then fixed in 100% methanol. Parasites were stained with rabbit anti-SAG1 antisera followed by AlexaFuor594-goat-anti-rabbit. The number of attached parasites was imaged and quantified as described above. Results are representative of data from at least three independent experiments.

### Rhoptry secretion assay

Toxoplasma SeCreEt parasites were used to infect Cre-reporter host cells essentially as described [31] with minor modifications. Extracellular SeCreEt parasites were prepared and treated with the GST proteins as described above. Approximately 30 minutes following invasion, infected monolayers were washed three times in complete media and then incubated at 37°C for 24 hours in complete media supplemented with a buffer control, 5 µM GST, or 5 µM GST-D3. Infected monolayers were washed once in 1× PBS and then fixed in formaldehyde as described above. The number of GFP-positive host cells were counted in 15 randomly selected fields on each of four separately mounted coverslips for each condition and visualization was performed using a 20× lens on a Nikon Eclipse TE300 microscope. The number of infected or uninfected GFP-positive host cells was counted in randomly selected fields on each of four separately mounted coverslips for each condition and visualization was performed using a 100× oil-immersion lens on an Olympus BX60 upright fluorescent microscope. For each condition, 100 GFP-positive cells were examined. Results are representative of data from two independent experiments.

### Statistical analyses

The Graphpad Prism program was used to determine if the differences observed in the data for the invasion, attachment, and rhoptry secretion assays were statistically significant. Data sets were analyzed using the unpaired Student's *t*-test (two-tailed), under the assumption of equal variance. A *p*-value of less than 0.05 was considered to be statistically significant in the tests.

## Supporting Information

Figure S1 Pre-incubation of RH and RHΔ*ama1*/*AMA1-myc* parasites with GST-D3 decreases invasion efficiency in a dose-dependent manner. Extracellular RH (A) or RHΔ*ama1*/*AMA1-myc* (B) parasites were pre-treated with a buffer control, GST alone, or GST-D3 at a concentration of 5, 1.25, or 0.625 µM essentially as described in Figure 6. The number of intracellular parasites was determined for 15 randomly-selected fields from three coverslips for each condition tested. The invasion levels for each condition are shown relative to the buffer-treated control (shown are means with standard deviation). An asterisk indicates a statistically significant increase in invasion levels relative to the 5 µM GST-D3 treatment (unpaired Student's t-test), with p<0.0023 (A) or p<0.0012 (B).
(0.10 MB TIF)

Table S1 Primers used in these studies.
(0.06 MB DOC)
