# Supplementary figures and images for: The C-Terminus of Toxoplasma RON2 Provides the Crucial Link between AMA1 and the Host-Associated Invasion Complex
Source: PLoS Pathog. 2011 Feb 10;7(2):e1001282. doi: 10.1371/journal.ppat.1001282 (PMC3037364; doi:10.1371/journal.ppat.1001282)

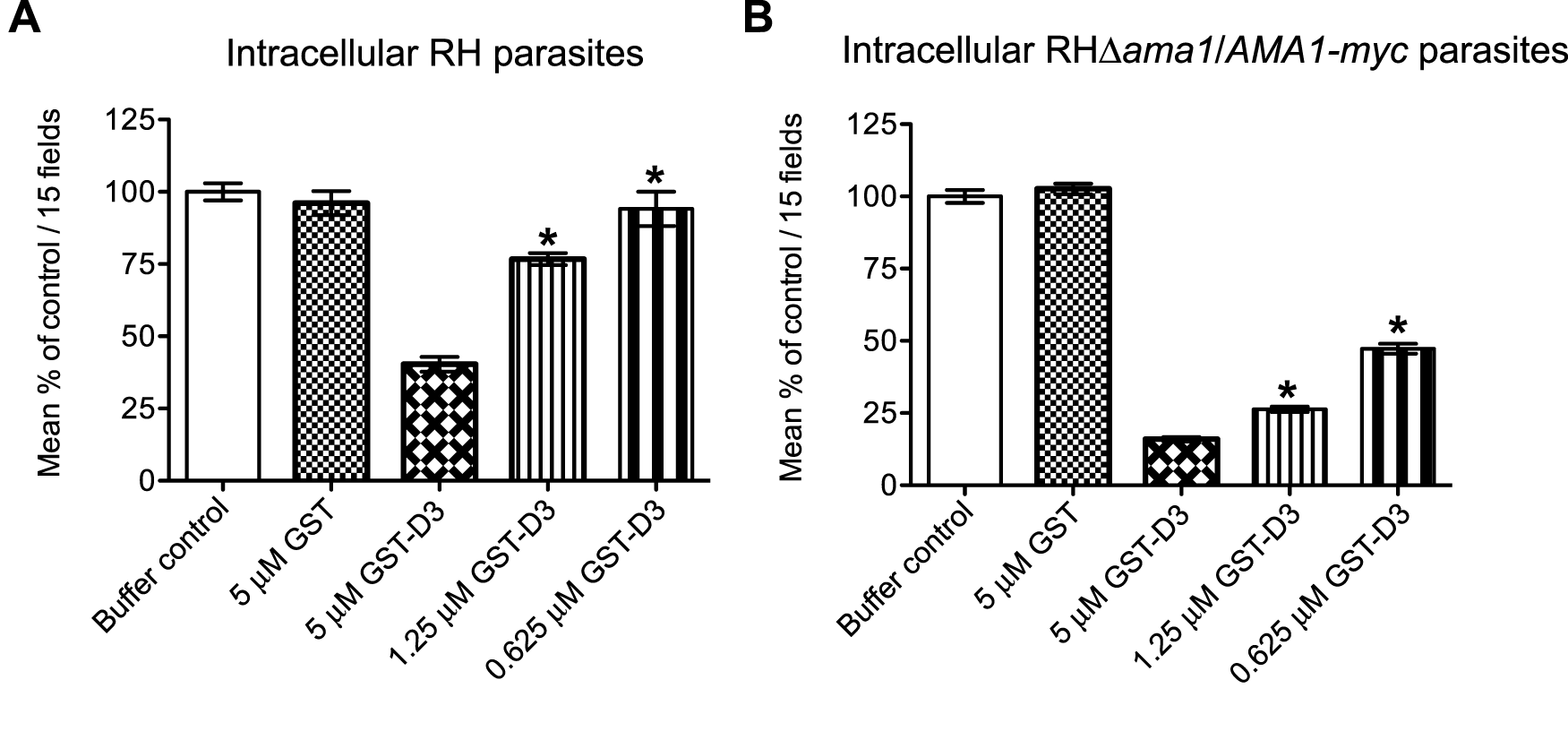

Supplement: Figure S1 — Pre-incubation of RH and RHΔama1/AMA1-myc parasites with GST-D3 decreases invasion efficiency in a dose-dependent manner. Extracellular RH (A) or RHΔama1/AMA1-myc (B) parasites were pre-treated with a buffer control, GST alone, or GST-D3 at a concentration of 5, 1.25, or 0.625 µM essentially as described in Figure 6. The number of intracellular parasites was determined for 15 randomly-selected fields from three coverslips for each condition tested. The invasion levels for each condition are shown relative to the buffer-treated control (shown are means with standard deviation). An asterisk indicates a statistically significant increase in invasion levels relative to the 5 µM GST-D3 treatment (unpaired Student's t-test), with p<0.0023 (A) or p<0.0012 (B). (0.10 MB TIF) [file ppat.1001282.s001.tif]
