# Supplementary material for: The C-Terminus of Toxoplasma RON2 Provides the Crucial Link between AMA1 and the Host-Associated Invasion Complex
Source: PLoS Pathog. 2011 Feb 10;7(2):e1001282. doi: 10.1371/journal.ppat.1001282 (PMC3037364; doi:10.1371/journal.ppat.1001282)
Supplement: Table S1 — Primers used in these studies. (0.06 MB DOC) [file ppat.1001282.s002.doc]

**Table S1. Primers used in these studies.**

| **Primer name** | **Sequence** | **Use** |
| --- | --- | --- |
| A | 5’- CTAGCTAGCACGACGGAGAAAACGTCCGAA-3’ | Amplifying 3’ targeting sequence for generating TgRON2-HA parasites |
| B | 5’- AAGGGCCCAAGTGCTCTGATCGCGTGTCT-3’ | Amplifying 3’ targeting sequence for generating TgRON2-HA parasites |
| C | 5’- GGGGTACCGCGTTTTCGTTCGGAGGCTGTAGA-3’ | Amplifying 5’ targeting sequence for generating TgRON2-HA parasites |
| D | 5’- CCGGATATCTTACGCGTAGTCCGGGACGTCGTACGG-GTAGGCTTTGATGAGAGGCGCAC-3’ | Amplifying 5’ targeting sequence for generating TgRON2-HA parasites |
| E | 5’-ACTCGGCGATGTTTCTTGACGCA-3’ | Confirmation of proper integration of *TgRON2-HA* at 5’ end |
| F | 5’-TAGTCCGGGACGTCGTACGGGTA-3’ | Confirmation of proper integration of *TgRON2-HA* at 5’ end |
| G | 5’-CAATGTAGGGCTTGCAGTG-3’ | Confirmation of proper integration of *TgRON2-HA* at 3’ end |
| H | 5’-TTGCATGCCCTCTCCCTGTTG -3’ | Confirmation of proper integration of *TgRON2-HA* at 3’ end |
| I | 5’- CGGGATCCCTGAATGTTGCAGACATCGT-3’ | Amplification of TgRON2 domain 3 |
| J | 5’-CGGAATTCTTAGACTTTGAGGAAGTCTTGGGT-3’ | Amplification of TgRON2 domain 3 |
| K | 5’-CGGGATCCTGGAACATCCTCAAGTCGGA-3’ | Amplification of TgRON2 domain 4 |
| L | 5’CGGAATTCTTAGGCTTTGATGAGAGGCGCAC-3’ | Amplification of TgRON2 domain 4 |
| M | 5’-CGGGATCCGTcGAcAAcACcGAcCAaGGaAAaGTg-GCaGTtTTcGCccTcGTctccGAaATcGTgAAgGTcagcGCcGGcCAaGAgGAcGCccTcTg-3’ | Amplification of GST-D3scramble |
| N | 5’-CGGAATTCTTAaATcGGgCCCATgGCtGCgATcGGc-GGcGCcGCcAgaTTaTGgGTcTGcGTaGTcACgGTgCAgATcGCTCTgCAgAggGCgTCcTCtT-3’. | Amplification of GST-D3scramble |
